# Small RNA Response to Infection of the Insect-Specific Lammi Virus and Hanko Virus in an *Aedes albopictus* Cell Line

**DOI:** 10.3390/v13112181

**Published:** 2021-10-29

**Authors:** Pontus Öhlund, Juliette Hayer, Jenny C. Hesson, Anne-Lie Blomström

**Affiliations:** 1 Department of Biomedical Sciences and Veterinary Public Health, Swedish University of Agricultural Sciences, P.O. Box 7028, 750 07 Uppsala, Sweden; anne-lie.blomstrom@slu.se; 2 Department of Animal Breeding and Genetics, Swedish University of Agricultural Sciences, SLU-Global Bioinformatics Centre, P.O. Box 7023, 750 07 Uppsala, Sweden; juliette.hayer@slu.se; 3 Department of Medical Biochemistry and Microbiology, Zoonosis Science Center, Uppsala University, P.O. Box 582, 751 23 Uppsala, Sweden; jenny.hesson@imbim.uu.se

**Keywords:** insect-specific flaviviruses, Hanko virus, Lammi virus, small interfering RNA, Piwi-interacting RNA

## Abstract

RNA interference (RNAi)-mediated antiviral immunity is believed to be the primary defense against viral infection in mosquitoes. The production of virus-specific small RNA has been demonstrated in mosquitoes and mosquito-derived cell lines for viruses in all of the major arbovirus families. However, many if not all mosquitoes are infected with a group of viruses known as insect-specific viruses (ISVs), and little is known about the mosquito immune response to this group of viruses. Therefore, in this study, we sequenced small RNA from an *Aedes albopictus*-derived cell line infected with either Lammi virus (LamV) or Hanko virus (HakV). These viruses belong to two distinct phylogenetic groups of insect-specific flaviviruses (ISFVs). The results revealed that both viruses elicited a strong virus-derived small interfering RNA (vsiRNA) response that increased over time and that targeted the whole viral genome, with a few predominant hotspots observed. Furthermore, only the LamV-infected cells produced virus-derived Piwi-like RNAs (vpiRNAs); however, they were mainly derived from the antisense genome and did not show the typical ping-pong signatures. HakV, which is more distantly related to the dual-host flaviviruses than LamV, may lack certain unknown sequence elements or structures required for vpiRNA production. Our findings increase the understanding of mosquito innate immunity and ISFVs’ effects on their host.

## 1. Introduction

Most of our current knowledge on virus interactions with the mosquito vector has come from studies on human pathogenic arboviruses such as West Nile virus (WNV), Dengue virus (DENV), and Chikungunya virus (CHIKV). However, mosquitoes are often naturally and persistently infected with a group of viruses that are known as insect-specific viruses (ISVs). This group of viruses are unable to infect vertebrates and are maintained in the mosquito population through vertical transmission from mother to offspring [1,2,3,4]. Moreover, phylogenetic analyses have shown that many ISVs belong to viral families associated with pathogenic arboviruses such as *Flaviviridae, Togaviridae,* and *Peribunyaviridae* [5,6]. Insect-specific flaviviruses (ISFVs) can further be divided into two distinct phylogenetic subgroups. Those of the first subgroup are phylogenetically distinct from all other known flaviviruses and are referred to as classical ISFVs. The ISFVs of the other subgroup are phylogenetically affiliated with the medically important dual-host flaviviruses but have the insect-restriction phenotype and are referred to as dual-host affiliated ISFVs [7]. Two members of the insect-specific flavivirus group are Lammi virus (LamV) and Hanko virus (HakV), which were both isolated from mosquitoes in Finland. LamV was isolated from *Aedes cinereus* mosquitos and belongs to the dual-host affiliated ISFV category [8], and HakV was isolated from *Aedes caspius* mosquitoes and belongs to the classical ISFV category [9]. Little is known about how these ISFVs interact with the mosquito and whether they trigger an antiviral immune response in the host.

The main antiviral immune response in mosquitoes is believed to be the RNA interference (RNAi) pathway, which includes three major classes of regulatory small RNAs (sRNAs): Small interfering RNA (siRNA), microRNA (miRNA), and P-element-induced wimpy testis (Piwi)-interacting RNA (piRNA) [10]. The siRNA pathway is possibly the most important one with regards to antiviral defense in the mosquito. Upon a viral infection, double-stranded RNA (dsRNA) will form as replicative intermediates in the cytoplasm. These foreign (exogenous) dsRNAs are recognized and processed by the Dcr2–R2D2 complex into siRNAs of 21 nucleotides (nt) in length. Furthermore, the siRNA is incorporated into the RNA-induced silencing complex (RISC), where one of the strands is degraded and the remaining guide strand is used as a template to recognize complementary target RNAs, which are subsequently cleaved and degraded by Ago2 [11,12,13]. Functional Dcr2, R2D2, and Ago2 are crucial for limiting virus replication and dissemination in the mosquito, as demonstrated by many studies [14,15,16,17,18].

The miRNA pathway is similar to the siRNA pathway in the sense that dsRNAs are processed into smaller dsRNAs that are loaded into an RISC complex and facilitate target-specific cleavage. In the miRNA pathway, Dcr1 and Ago1 are analogous to Dcr2 and Ago2, respectively, in the siRNA pathway. However, the primary function of the miRNA pathway is post-transcriptional gene regulation, and the miRNAs (20–25 nt) come from endogenous hairpin transcripts [19,20]. The miRNA pathway has been shown to play a critical role in modulating host genes to control viral infection, e.g., the Aae-miR-2940-5p miRNA was found to be selectively downregulated in C6/36 cells as a response to WNV infection. This resulted in a lower expression of the metalloprotease m41 FtsH gene, which has been shown to be important for WNV replication [21]. Other studies have observed miRNA-driven gene modulation during the active viral infection of Zika virus (ZIKV) [22], DENV [23], and WNV [24].

The primary roles of the piRNA pathway are to silence transposons and maintain the integrity of the germline [25]. The key proteins of this pathway are members of the PIWI and Argonaut superfamilies, and the *Aedes albopictus* genome is known to encode seven PIWI proteins (Piwi 1–6 and Ago3) [26]. The piRNAs are 24–30 nt in length and usually show specific signatures features such as an uracil (U) bias at the first position of the antisense strand and an adenine (A) at the 10th position of the sense strand. Recent studies on mosquitoes have suggested a possible role of the piRNA pathway in antiviral defense. For example, the production of ping-pong-dependent virus-derived piRNAs (vpiRNAs) by the PIWI-5 and Ago3 proteins has been observed during viral infection, suggesting an antiviral function [27]. Furthermore, the knockdown of the PIWI-4 proteins in mosquito cell lines has been found to increase the viral titers in comparison to controls [28,29,30]. The exact antiviral mechanisms of the piRNA pathway are still unknown.

In this study, we infected the *Aedes albopictus* U4.4 cell line with either of two ISFVs from two distinct phylogenetic groups, LamV or HakV, to investigate the small RNA response. We found that both LamV and HakV elicited a strong virus-derived small interfering RNA (vsiRNA) response that increased over time and targeted the whole viral genome. However, only the LamV-infected cells produced viral pi-like RNAs (vpi-like RNAs), which were mainly derived from the antisense genome and did not show the characteristic ping-pong signatures.

## 2. Materials and Methods

### 2.1. Cell Culture

*Aedes albopictus* U4.4 cells (kindly provided by Associate Professor G. Pijlman, Wageningen University, Wangeningen, The Netherlands) and C6/36 cells (Sigma-Aldrich, Darmstadt, Germany) were cultured at 28 °C in a Leibovitz L-15 medium (Gibco, Paisley, UK) supplemented with 10% fetal bovine serum (FBS) (Gibco, Paisley, UK), 10% tryptose phosphate broth (TPB) (Gibco, Paisley, UK), amphotericin (250 μg/mL) (Gibco, Grand Island, NY, USA), and Pen Strep (100 U penicillin/mL and 100 μg streptomycin/mL) (Gibco, Grand Island, NY, USA).

### 2.2. Virus Stocks and Virus Titration

The ISFVs LamV (Strain: 2009/FI/Original) and HakV (Strain: 2005/FI/UNK) were obtained from the European Virus Archive—Global (EVAg). Virus stocks were propagated in C6/36 *Aedes albopictus* cells until a clear cytopathic effect (CPE) was shown (4 days post infection (d.p.i.)), and then supernatant was collected, centrifuged, and frozen at −80 °C. We did not obtain any reliable virus stock titers with the traditional methods. Thus, to quantify the ISFV stocks, plasmid standards containing the PCR target region of the virus were ordered (GeneScript Biotech, Leiden, The Netherlands). The plasmids were used to construct a qPCR standard curve, and a stock concentration was calculated as RNA copies/μL. RNA extractions and qRT-PCR protocols are described below.

### 2.3. In Vitro Infection

On the day of infection, U4.4 cells grown in 24-well plates to a confluence of 85–90% were used (approximately 350,000 cells per well). The cells were infected as triplicates for each timepoint. In brief, per infection, 35,000 RNA copies of either LamV or HakV were mixed with 200 μL of infection medium (Leibovitz L-15 medium containing 2% FBS and 10% TPB) and added to the respective wells. After one hour of incubation at 28 °C, the inoculum was discarded, and 500 μL of Leibovitz L-15 medium supplemented with 5% FBS, 10% TPB, and PEST were added. Mock infected cells were used as controls. The U4.4 cells were sampled every 24 h until 72 h post-infection (p.i.) for small RNA sequencing, and the supernatant was collected from 24 to 96 h p.i. for qPCRs.

### 2.4. RNA Extractions

The RNA used for growth curves and virus titration was extracted from 200 μL of supernatant/virus stocks in 750 μL of TRIzol™ (Thermo Fisher Scientific, Carlsbad, CA, USA) and homogenized. The aqueous phase obtained after the addition of chloroform and the subsequent centrifugation step was collected, diluted to 1:1 with freshly prepared 70% ethanol, and purified with GeneJet spin columns (Thermo Fisher Scientific, Vilnius, Lithuania). The RNA was eluted in 40 μL of nuclease-free water and stored at −80 °C until further processing.

Small RNA used for high-throughput sequencing was isolated from cells that were collected by adding 750 μL of TRIzol™ to respective wells. The aqueous phase was obtained in the same manner as described above, and the RNA was further purified using the mirVana™ PARIS™ Kit (Thermo Fisher Scientific, Vilnius, Lithuania) according to the protocol to isolate small RNA (<200 nt) provided by the manufacturer.

### 2.5. qPCR

First-strand cDNA was generated using the SuperScript™ III Reverse Transcription kit (Thermo Fisher Scientific, Carlsbad, CA, USA) with Random Hexamers (Thermo Fisher Scientific, Carlsbad, CA, USA) following the manufacturer’s instructions, with an input of 5 μL of RNA in a total reaction volume of 20 μL. The qPCR was performed using the iTaq Universal SYBR^®^ Green Supermix (Bio-Rad laboratories Inc., Hercules, CA, USA) with 2 μL of template cDNA and 0.5 μM of each corresponding virus primer (Table 1) in a total volume of 20 μL per reaction. The qPCR was carried out using the Bio-Rad^®^ CFX96 real-time PCR system (Bio-Rad laboratories Inc., Hercules, CA, USA), with amplification conditions consisting of an initial denaturation at 95 °C for 30 s, followed by 40 cycles of denaturation at 95 °C for 7 s, and finally annealing/extension and plate reading at 60 °C for 30 s. A melt curve was generated starting at 60 °C with a 0.5 °C increase up to 96 °C.

Primer pairs for the qPCR were designed using the software Primer3 [31] to generate a product between 170 and 200 bp long and a Tm of 60 °C. Virus reference genomes were obtained from the NCBI database and are listed together with the primer sequences in Table 1.

### 2.6. High-Throughput Sequencing of Small RNA

Small RNA isolated from the infected U4.4 cells was quantified and quality-controlled with the 4150 TapeStation System using the RNA ScreenTape Analysis kit (Agilent Technologies, Santa Clara, CA, USA). Triplicates for each timepoint (24–72 h) were pooled and submitted to SciLifeLab (Stockholm, Sweden) for library preparation using the QIAseq miRNA low input kit (QIAGEN, Hilden, Germany), and libraries were sequenced on one flow cell of the NextSeq 2000 with a 101nt(Read1)-8nt(Index1) setup using the ‘P2′ flow cell, which generated 10–20 million reads per sample with a read length of 1 × 100 base-pairs (bps).

### 2.7. Small RNA Sequence Analysis

The generated small RNA sequence data (FASTQ files) were analyzed with a pipeline including the trimming of adaptors and the removal of bad quality reads using Trim Galore! (v0.6.6), with a setting to discard all reads below 17 nt and above 40 nt. The trimmed reads were mapped to the LamV (FJ606789) genome and the HakV (NC030401) genome using Bowtie (v1.2.3) [32], allowing for one mismatch. We used the -a, --best, –strata, and --all flags, which instructed Bowtie to only report those alignments in the best alignment stratum and to generate a FASTQ file of the mapped reads in addition to the SAM file. The FASTQ files were used to analyze the size distribution of the mapped reads, which was visualized with the R package ggplot2. The SAM output files were further analyzed with the MISIS-2 software (v2.6) to visualize the alignment and polarity distribution of small RNA to the viral genomes [33]. The ping-pong signatures of 27–30 nt piRNA were analyzed with WebLogo [34].

### 2.8. Data Availability

All sequencing data have been made publicly available at the NCBI Sequence Read Archive (SRA) under accession number PRJNA761074.

## 3. Results

**Figure 1:**
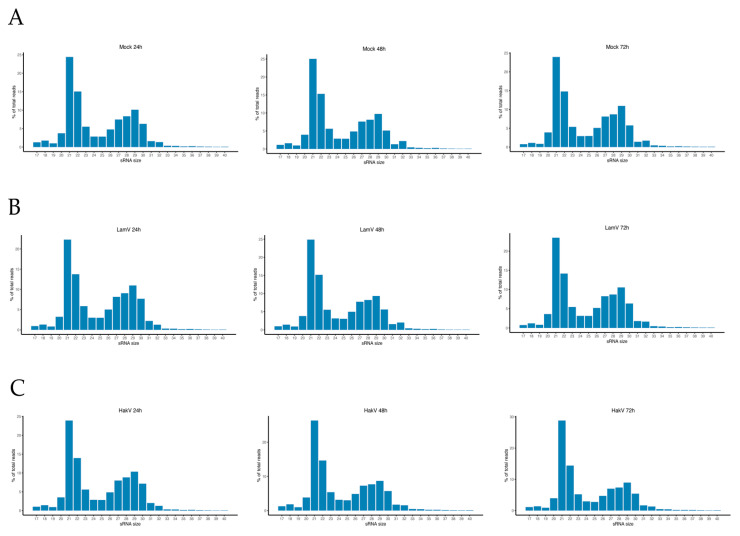
Small RNA profiles of U4.4 cells at 24, 48, and 72 h. (**A**) Small RNA profile of total reads in mock infected cells; (**B**) small RNA profile of total read in LamV-infected cells; (**C**) small RNA profile of total reads in HakV-infected cells.

To investigate the RNAi response to ISFV infection in mosquitoes, we infected the *Aedes albopictus* U4.4 cell line with LamV or HakV at a concentration of 175,000 RNA copies/mL. Mock infections were used as controls. Cells were collected at 24, 48, and 72 h p.i. and processed for small RNA sequencing. The retrieved sequence data were trimmed from adapters and bad quality reads, resulting in 10.4–16.9 million reads per sample, with read lengths between 17 and 40 nt. The size distributions of small RNAs were similar between all samples, with the highest peak observed at 21 nt (22.3–26.3% of total reads) and the second highest peak observed at 22 nt (13.7–15.3% of total reads) (Figure 1). These two peaks most likely corresponded to the populations of siRNAs and miRNAs in the cell. We could also observe an elevation in the proportion of reads between 26 and 30 nt, which is in the size range of piRNAs (Figure 1).

**Figure 2:**
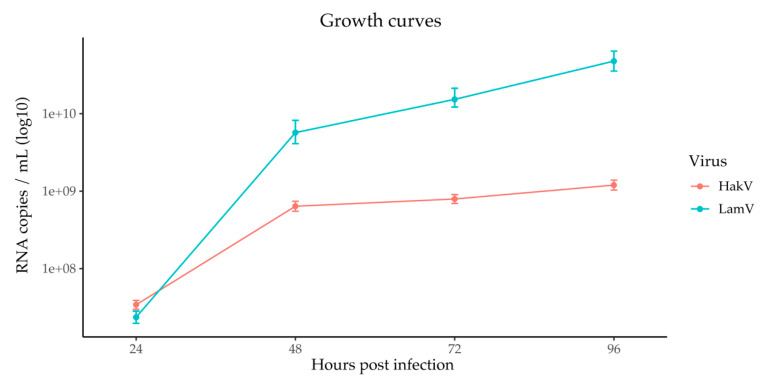
Growth curves of LamV and HakV in U4.4 cells, shown as RNA copies over time. Dots show the mean RNA copy numbers with a standard deviation between the biological triplicates.

To confirm active viral infection and replication, the supernatant was collected, further processed by RNA extraction and cDNA synthesis, and analyzed with qPCR. The data were used to calculate a growth curve over time (Figure 2). Both LamV and HakV showed steep virus growth during the first 48 h before plateauing. Furthermore, LamV showed a higher replication compared to HakV, with approximately one log more RNA copies at 48 h p.i. and the later timepoints recorded (Figure 2).

### 3.1. Viral Small RNA Profiles in U4.4 Infected with LamV

**Figure 3:**
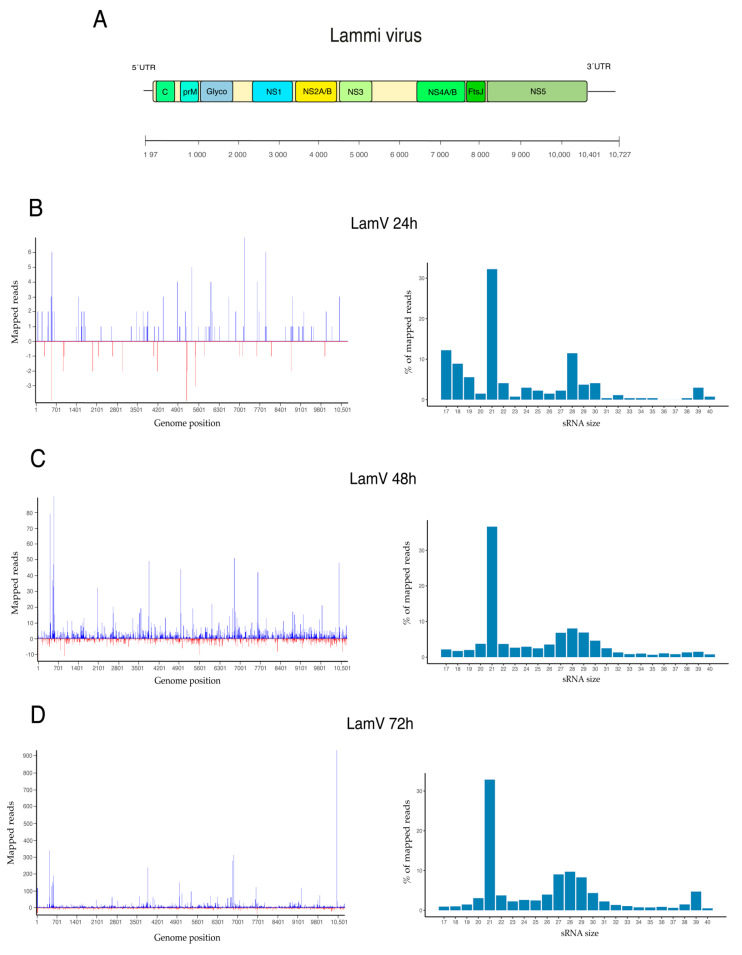
Viral small RNA profiles of U4.4 cells after LamV infection. (**A**) Schematic representation of the LamV genome and vsRNA profiles at 24 h (**B**), 48 h (**C**), and 72 h (**D**) p.i. Figures to the left show vsRNA reads along the LamV genome as a histogram. The positive values (blue) are counts of sRNAs mapped to the sense strand, and the negative values (red) are those mapped to the antisense strand. Figures to the right show the size distribution of reads that mapped to the LamV genome.

**Figure 4:**
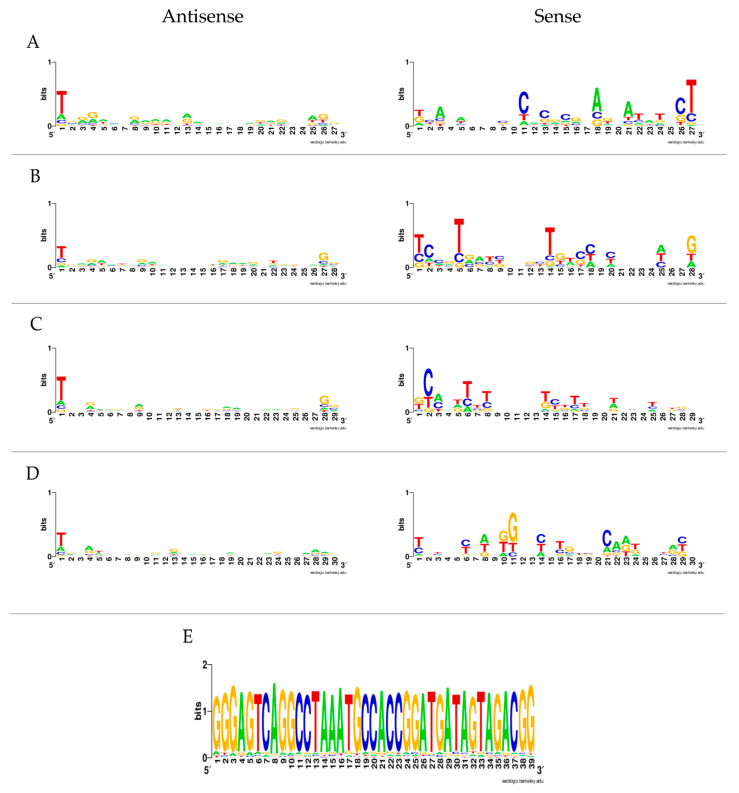
Nucleotide biases of the 27–30 and 39 nt long reads at 72 h p.i. of LamV. The sequences are in DNA format, so U is written as T. Reads derived from the antisense are shown in the left panel. Reads derived from sense strand are shown in the right panel: (**A**) 27 nt reads, (**B**) 28 nt reads, (**C**) 29 nt reads, (**D**) 30 nt reads, and (**E**) 39 nt long reads from the fourth hotspot in the LamV genome.

To further investigate the production of virus-derived small RNAs (vsRNAs), the trimmed sequence data were mapped to the viral genomes. Results from U4.4 cells infected with LamV showed that few reads were virus-specific at 24 h p.i.: only 0.001% (*n* = 270) of the reads mapped to the LamV genome. However, the proportion of virus-specific reads increased over time, with 0.06% (*n* = 6096) observed at 48 h p.i. and 0.17% (*n* = 24,179) observed at 72 h p.i. The majority of the mapped reads had a read length of 21 nt and most likely corresponded to vsiRNA (32.2% at 24 h, 36.7% at 48 h, and 32.9% at 72 h) (Figure 3). At 48 and 72 h p.i. a shoulder of reads in the size range of 27–30 nt that resembled vpiRNA was observed (Figure 3c,d). These 27–30 nt long reads were examined for ping-pong signatures, with antisense piRNA having a 1U bias (shown as T in this dataset) and the sense piRNA having a 10A bias. The 1U characteristics for Piwi-5-bound piRNAs were identified; however, the 10A bias was not observed (Figure 4a–d).

The vsRNAs from the LamV-infected cells mapped across the entire LamV genome, with a few predominant hotspots with a higher coverage. These hotspots were most distinct at 72 h p.i. (Figure 3d) and included four positions in the genome. Starting from the 5′ end of the LamV genome, the first hotspot was at position 423, which was in the gene encoding for the capsid protein. The second hotspot was at position 3854, i.e., in the NS2A gene. The third hotspot at position 6808 was located in the NS4A protein, and the fourth hotspot at position 10,437 was positioned in the 3′-UTR of the genome. Moreover, the fourth hotspot had a coverage of over 900 reads, mainly at the size of 39 nt, which could been seen as a small hump in the read length distribution (Figure 3d). These reads were extracted and analyzed with WebLogo, and it was found that the majority consisted of the same sequence (Figure 4e). This suggested that these 39 nt long reads were not degraded products; however, their function and biogenesis are uncertain. Interestingly, a large majority of the vsiRNA mapped to the sense strand of the LamV genome: 79.2% at 48 h and 82.4% at 72 h p.i. (Figure 3, left panel). This suggests that Ago-2 has a preferred bias for the antisense strand as a template and that the RISC complex mainly targets and degrades the sense genomes during LamV infection.

### 3.2. Viral Small RNA Profiles in U4.4 Infected with HakV

**Figure 5:**
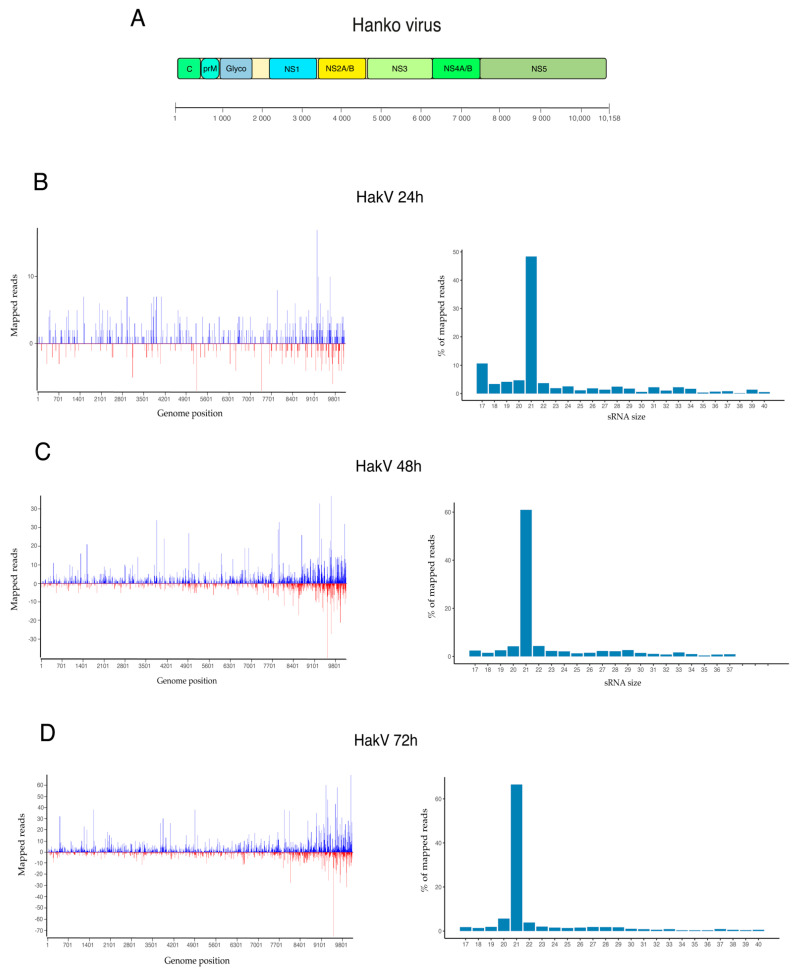
Viral small RNA profiles of U4.4 cells after HakV infection. (**A**) Schematic representation of the HakV genome. vsRNA profiles at 24 h (**B**), 48 h (**C**), and 72 h (**D**) p.i. Figures to the left show vsRNA reads along the HakV genome as a histogram. The positive values are counts of vsRNAs mapped to the sense strand, and the negative values are those mapped to the antisense strand. Figures to the right show the size distribution of reads that mapped to the HakV genome.

The results from HakV-infected U4.4 cells showed a slightly higher production of vsRNAs at 24 h p.i. compared to LamV-infected cells, where 0.01% (*n* = 1296) of the reads mapped to the HakV genome. The observed proportion of vsRNAs was similar at 48 h p.i., 0.06% (*n* = 7324), and lower at 72 h p.i., 0.08% (*n* = 11,179) compared to LamV-infected cells. Moreover, when analyzing the read length distribution, a high proportion of the reads was in the size range of siRNA (21 nt), with 48.4% observed at 24 h p.i, 60.9% observed at 48 h p.i., and 66.5% observed at 72 h p.i. The shoulder of piRNAs (27–30 nt) observed in the LamV-infected cells was absent in those cells infected with HakV (Figure 5). The lack of vpiRNA suggests that HakV does not trigger the proteins responsible for vpiRNA amplification such as Ago3 and PIWI-5. An analysis of the read alignment showed that the vsRNA mapped across the entire HakV genome, with slightly more coverage observed at the 3′ end (7840–10,158 nt), which corresponded to the RNA-dependent RNA polymerase (NS5) gene region. The strand polarity showed a more even distribution between sense (68.6%) and antisense (31.4%) compared to LamV-infected U4.4 cells.

## 4. Discussion

RNAi-mediated antiviral immunity is thought to be key in the defense against viral infection in mosquitoes. The production of virus-specific small RNAs has been demonstrated in mosquitoes and mosquito-derived cell lines for viruses in all of the major arbovirus families: Peri*bunyaviridae*, *Togaviridae,* and *Flaviviridae* [18,35,36,37,38,39,40]. However, although mosquitoes and mosquito-derived cell lines are often persistently infected with this group of viruses, the RNAi response to ISVs is not as well-studied. To our knowledge, only a handful of studies have investigated the effect of ISVs on the RNAi response in mosquitoes. One study developed an integrated mosquito small RNA genomics resource and included data from mosquito cell lines persistently infected with ISVs, including the ISFV cell-fusing agent virus [41]. Another study investigated the RNAi response of the mosquito U4.4 (*Aedes albopictus*), Aag2 (*Aedes aegypti),* and CT (*Culex tarsalis*) cell lines, which were shown to be persistently infected with different ISVs. For example, the U4.4 cells were persistently infected with Culex Y virus (CYV) and displayed a potent siRNA response against it [42]. In the present study, we investigated the RNAi response to acute ISFV infection over time in the aforementioned U4.4 cells. At the early time point (24 h p.i.), both the LamV- and HakV-infected U4.4 cells showed low amounts of vsRNAs (0.01–0.001% of total reads), which could have been because of the relatively low amount of virus or because the siRNA and piRNA pathways had not yet responded. Results from the qPCR analysis of the supernatant showed the replication of both LamV and HakV at this time point (Figure 2). Moreover, the proportion of vsRNA steadily increased over time, with 0.06% observed at 48 h p.i. and 0.17–0.08% observed at 72 h p.i. Interestingly, the increasing proportion of vsRNA correlated with the plateau of the virus growth curves (Figure 2) and might have been a sign of RNAi-induced interference with the virus replication.

The siRNA pathway is regarded as the most important antiviral defense mechanism, and in line with this, our experiments showed that it was the most abundant population of sRNAs between all groups and time points. Hence, this was observed in the general sRNA distribution—including the mock infected cells, most likely due to the persistent CYV infection (Figure 1)—and in the distribution of ISFV-specific sRNAs (Figure 3 and Figure 5). The LamV-infected cells had proportions of between 32.2 and 36.7% of vsiRNAs, and the HakV-infected cells had slightly higher proportions of between 48.4 and 66.5%. The vsiRNAs from LamV-infected cells mapped along the whole genome, with no particular coldspots but one predominant hotspot that correspond to the NS4AB proteins (Figure S1). We also observed that most of the vsiRNAs mapped to the sense strand of the genome, which could be a strategy for the efficient restriction of virus replication. The vsiRNAs of HakV-infected cells also mapped to the whole genome, with a slightly higher coverage at the 3′ end (7840–10,158 nt) corresponding to the gene coding for the NS5 protein (Figure S1). The strand polarity distribution was close to even, with slightly more vsiRNAs mapping to the sense strand. Although the siRNA response is regarded as the main antiviral immune response, it has also been postulated that siRNA is necessary for a persistent viral infection in mosquitoes [43,44]. The hypothesis behind this is that the siRNA response keeps the viral load in the host cell at a tolerable level, thereby sustaining a persistent infection. Many of the ISVs have been shown to not only persistently infect mosquitoes but also vertically transmit from mother to offspring [2,3,4]. Our data showed that the mosquito U4.4 cell line is able to elicit a strong virus-specific siRNA response to the studied ISFVs, which could support lifelong infection in mosquitoes.

In the LamV-infected cells, we observed a hotspot positioned in the 3′-UTR with a coverage of 900 reads, mainly ay the size of 39 nt. These reads mainly consisted of the same sequence (5′GGGAGTCAGGCCTAAATGCCACCGGATGATAGTAGACGG), suggesting that the reads were not degraded products (Figure 4e). The BLASTn search of the consensus sequence showed that this sequence could also be found in the 3′-UTR of another ISFV named Chaoyang virus (NC 017086), indicating that it may be a conserved sequence that can exist in the UTR of other ISFVs as well. However, the origin, function, and biogenesis of these 39 nt reads are uncertain.

Apart from the detection of vsiRNAs, putative vpiRNAs were identified in the LamV-infected cells. Earlier studies in an *Aedes aegypti* cell line have shown that the production of ping-pong-dependent vpiRNA relies on the PIWI-5 and Ago-3 proteins. The knockdown of either of these proteins in the Aag2 cell line infected with Sindbis virus or DENV-2 showed a massive decrease in vpiRNAs [27,45]. The ping-pong amplification of piRNAs is a two-step amplification mechanism where the PIWI-5 protein, loaded with a primary piRNA, cleaves a complementary target RNA. The 3′cleavage product is transferred to the protein Ago-3 and used as a template to cleave a complementary target RNA. This generates a new piRNA precursor for the Piwi-5 protein, which gives rise to the same primary piRNA sequence that initiated the amplification. Therefore, this type of amplification predicts an even distribution of piRNAs derived from both strands [27,46].

In the data from our U4.4 cells infected with LamV, we did not observe equal amounts of vpiRNA from both strands. The majority of the putative vpiRNAs (26–30 nt) were derived from the antisense strand, with the ping-pong characteristic nucleotide bias of an U at the first position. However, the few putative vpiRNAs derived from the sense strand did not show the nucleotide bias of an A in the tenth position characteristic for Ago-3 bound piRNA. Moreover, because of the unequal strand distribution, we could not analyze the overlap probability and look for the 10 nt overlap, which is significant for ping-pong-amplified piRNA (Figure 3 and Figure 4 and Figure S2). Similar observations have been made in studies where U4.4 cells were infected with WNV [42] and where Aag2 cells were infected with ZIKV [47]. In these studies, reads in the size range of 25–30 nt mainly mapped to the sense strand but with no 1U bias. These observations suggest that the identified putative LamV vpiRNAs are not ping-pong-dependent, and we hypothesize that they could be primary vpiRNAs cleaved by an ortholog to the zucchini proteins in *Drosophila* or some unknown endonuclease using either exogenous viral RNA or pre-primary RNA transcribed from viral-derived cDNA (vDNA) [30,48]. Further evaluation is needed to understand their function and biogenesis, as well as whether they interact with any of the PIWI proteins. Surprisingly, U4.4 cells infected with HakV did not elicit any production of these putative vpiRNAs (Figure 5), which suggests that the production of vpiRNAs is virus-dependent. HakV belongs to the group of classical ISFVs that are very distinct from all known flaviviruses [7]. It would be interesting to further investigate whether other viruses in the classical ISFV group show similar results, which could reveal factors important for putative vpiRNA production. We speculate that virus-specific sequence elements or structures trigger the production of vpiRNAs by an unknown Piwi-dependent amplification mechanism.

## 5. Conclusions

In conclusion, we showed that two ISFVs from two distinct phylogenetic groups can trigger the RNAi response during an acute infection of the *Aedes albopictus* U4.4 cell line. Both viruses elicited a strong vsiRNA response that increased over time and targeted the whole viral genome. Furthermore, infection with LamV (which is closely related to the pathogenic dual-host flaviviruses) triggered the production of putative primary piRNAs, while infection with HakV, which is more distantly related, did not. This suggests that the mosquito piRNA pathway is virus-specific and might need specific sequence elements or structures. These results contribute to our understanding of mosquito antiviral immunity, small RNA machineries, and how ISVs affect mosquitoes.

## Figures and Tables

**Table 1:** Primer pairs used for the qPCR analysis.

Primers	Binding Site	Sequence (5′ → 3′)	Ref
LamV-F	4659–4678	TGGGTGTTACCGGGTTATGT	FJ606789
LamV-R	4845–4864	ACGTTCCATTCAGTTTCCAT	
HakV-F	4655–4674	TGTGTTACGGTGGAAACTGG	NC030401
HakV-R	4842–4861	CAACTGGTTCTCCGTTGACA	

## Data Availability

All sequencing data have been made publicly available at the NCBI Sequence Read Archive (SRA) under accession number PRJNA761074.

## Supplementary Materials

The following are available online at https://www.mdpi.com/article/10.3390/v13112181/s1: Figure S1: Mapping of viral siRNA 72 h after ISFVs infection; Figure S2: Distribution of virus-derived piRNAs 72 h after LamV infection.
