# Supplementary material for: Small RNA Response to Infection of the Insect-Specific Lammi Virus and Hanko Virus in an Aedes albopictus Cell Line
Source: Viruses. 2021 Oct 29;13(11):2181. doi: 10.3390/v13112181 (PMC8620693; doi:10.3390/v13112181)
Supplement: Supplementary file 1 [file viruses-13-02181-s001.zip › viruses-1411382-supplementary final.pdf]

Supplementary Material

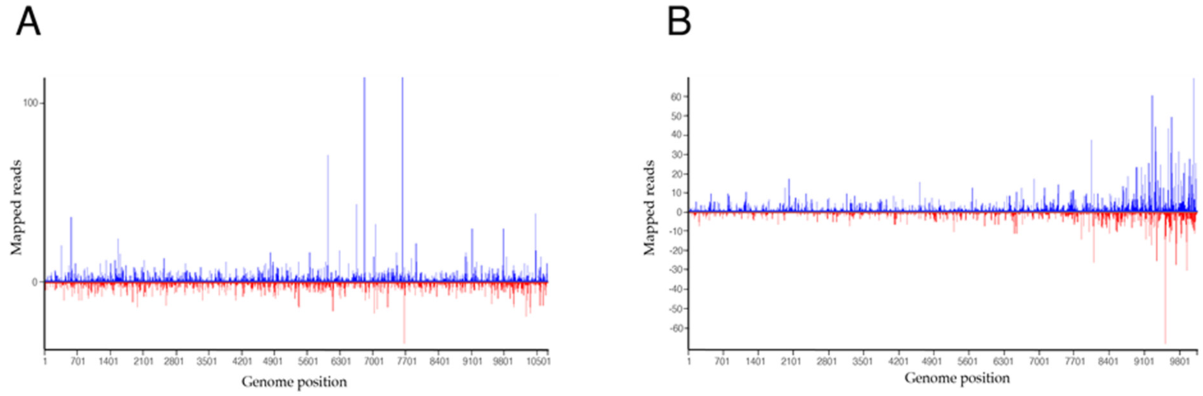

Figure S1. Mapping of viral siRNA at 72 h after ISFVs infection. (**A**) Distribution of 21 nt long reads to the LamV genome; (**B**) distribution of 21 nt-long reads to the HakV genome. The positive values are counts of vsiRNAs mapped to the sense strand, and the negative values are those mapped to the antisense strand.

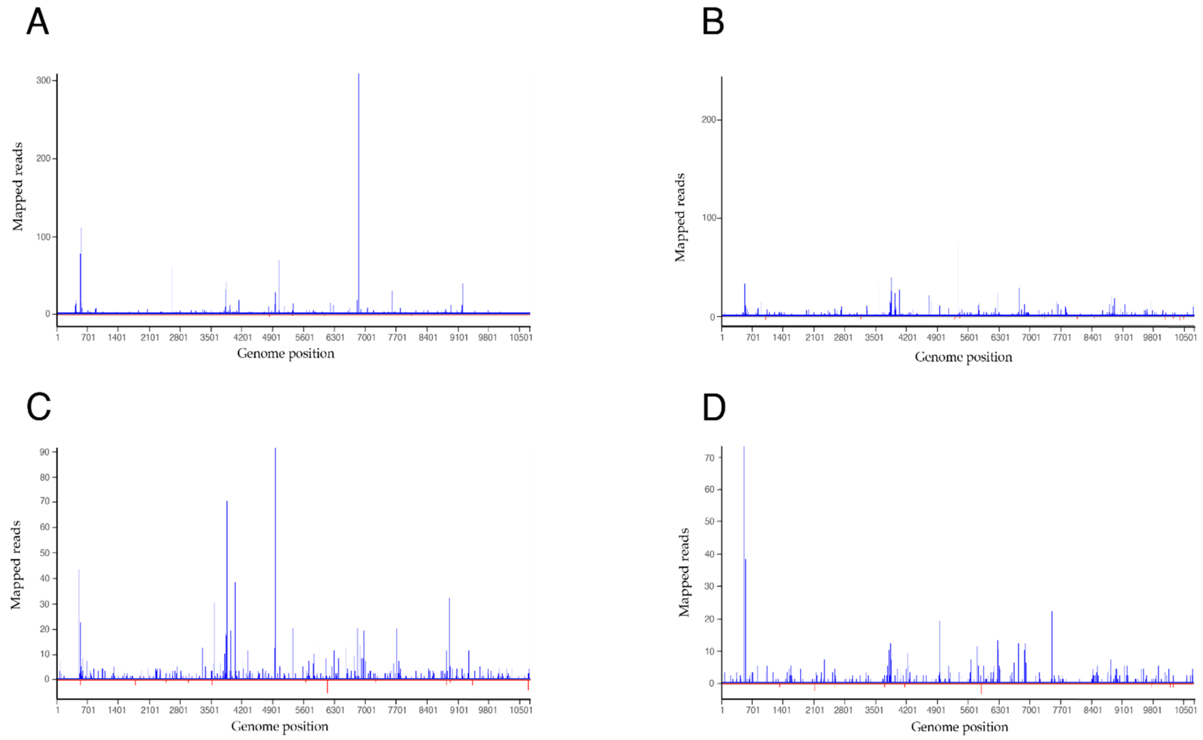

Figure S2. Distribution of virus-derived piRNAs of U4.4 cells at 72 h after LamV infection. (**A**) vpi-like RNA of 27 nt, (**B**) vpi-like RNA of 28 nt, (**C**) vpi-like RNA of 29 nt, and (**D**) vpi-like RNA of 30 nt. The positive values are counts of vpi-like RNAs mapped to the sense strand, and the negative values are those mapped to the antisense strand.
