# Supplementary figures and images for: Small RNA Response to Infection of the Insect-Specific Lammi Virus and Hanko Virus in an Aedes albopictus Cell Line
Source: Viruses. 2021 Oct 29;13(11):2181. doi: 10.3390/v13112181 (PMC8620693; doi:10.3390/v13112181)

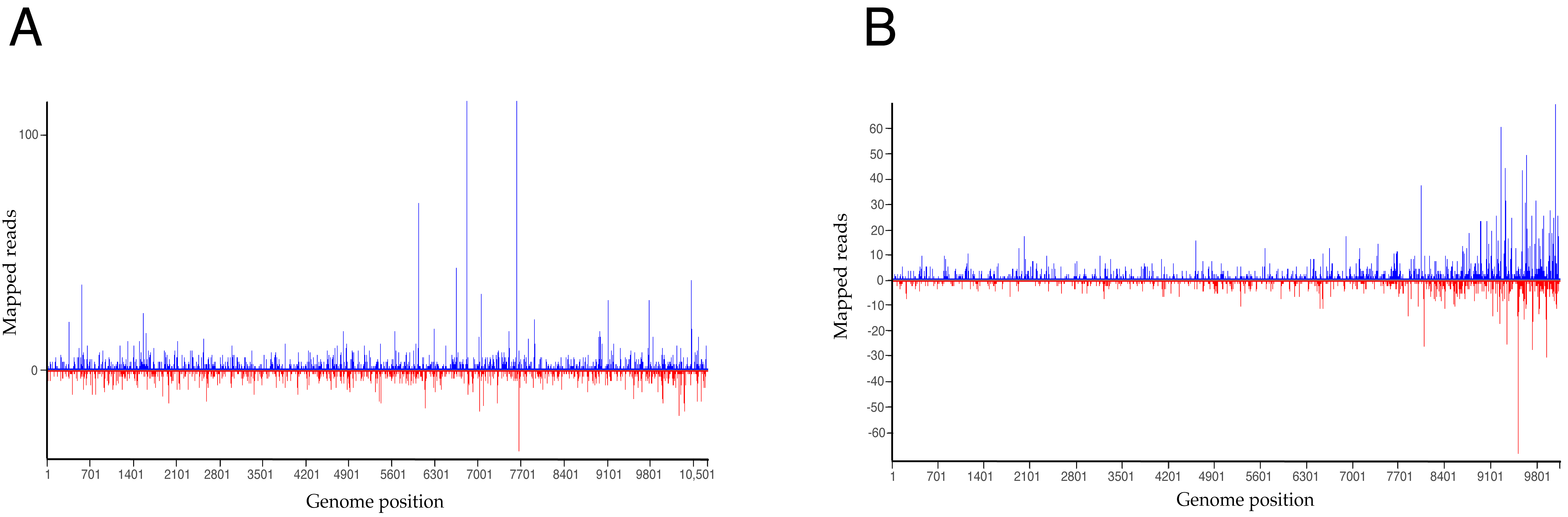

Supplement: Supplementary file 1 [file viruses-13-02181-s001.zip › Figure_S1.png]

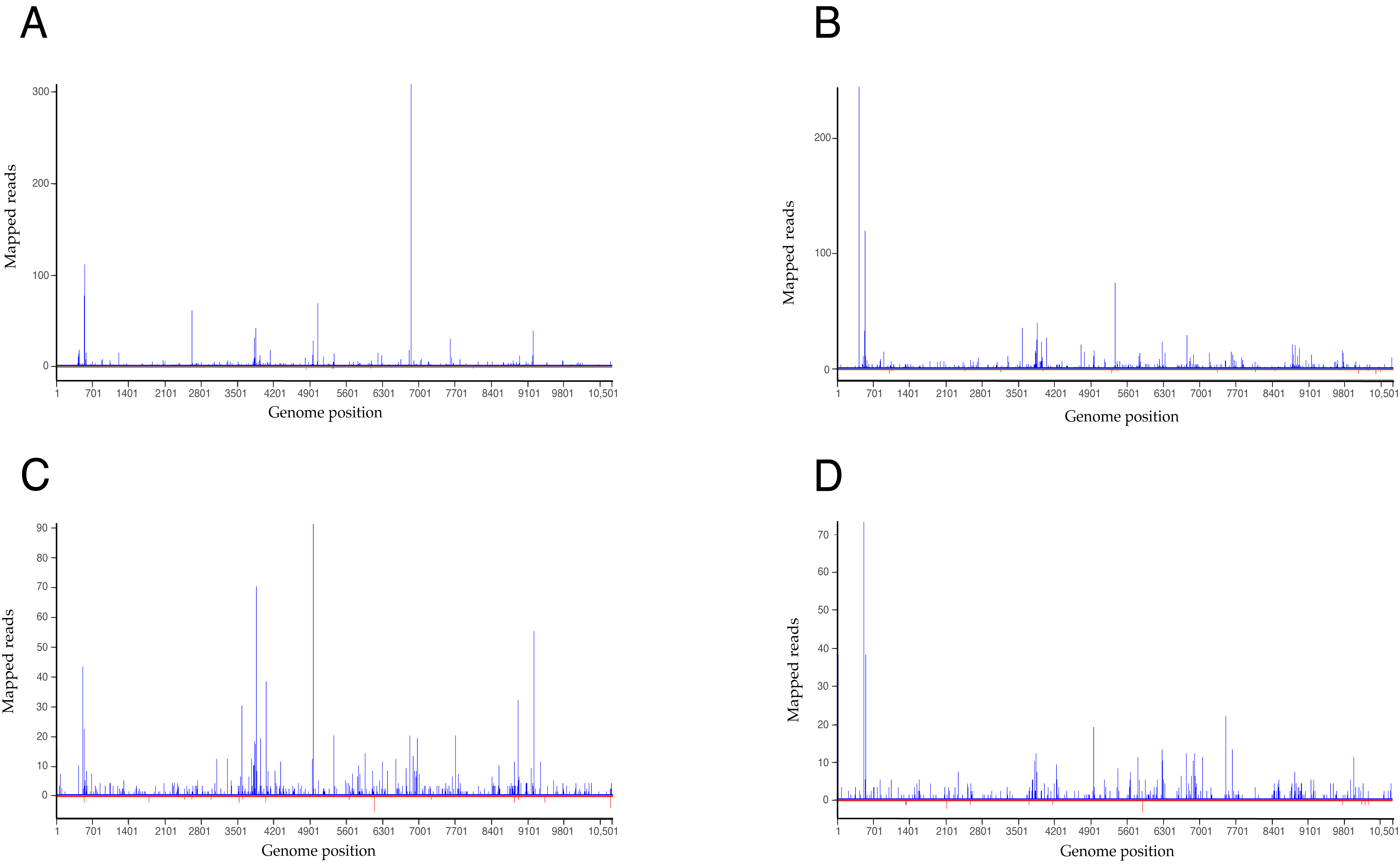

Supplement: Supplementary file 1 [file viruses-13-02181-s001.zip › Figure_S2.png]
